# Estimating thresholds of nitrogen, phosphorus and potassium fertilizer rates for rice cropping systems in China

**DOI:** 10.3389/fpls.2024.1470774

**Published:** 2024-09-12

**Authors:** Yingxia Liu, Wencheng Ding, Ping He, Xinpeng Xu, Wei Zhou

**Affiliations:** ^1^ Ministry of Agriculture and Rural Affairs Key Laboratory of Plant Nutrition and Fertilizer, State Key Laboratory of Efficient Utilization of Arid and Semi-arid Arable Land in Northern China, Institute of Agricultural Resources and Regional Planning, Chinese Academy of Agricultural Sciences, Beijing, China; ^2^ State Key Laboratory for Managing Biotic and Chemical Threats to the Quality and Safety of Agro-products/Institute of Environment, Resource, Soil and Fertilizers, Zhejiang Academy of Agricultural Sciences, Hangzhou, China

**Keywords:** fertilization thresholds, method for fertilizer demand, straw return, organic substitution, slow-release N

## Abstract

Determining the fertilization rate plays a pivotal role in agronomic practices as they directly impact yield targets, soil fertility, and environmental risks. In this study, we proposed a method that utilizes allowed ranges of partial nutrient balance and yield to estimate the threshold of nitrogen (N), phosphorus (P), and potassium (K) fertilizer applied to rice (*Oryza sativa* L.) fields in China. Based on a dataset of 6792 observations from rice fields, we determined the minimum and maximum rates of N, P and K suggested for single (mono-season rice), middle (summer-season rice rotated with winter-season upland crop), early and late (double-season rice cropping system) rice, ranging between 114−146 and 220−292 kg N ha^−1^ per season, 56−74 and 112−149 kg P_2_O_5_ ha^−1^ per season, and 170−230 and 329−347 kg K_2_O ha^−1^ per season, respectively. These values serve as the lower and upper fertilization thresholds, guiding yield goals and environmental protection. Furthermore, if rice straw is returned to fields, the demand for K fertilizer can theoretically decrease by 183 kg K_2_O ha^−1^, with corresponding decreases of 50 kg N ha^−1^ and 26 kg P_2_O_5_ ha^−1^, respectively. A recommended fertilization approach, excluding returned straw nutrients from the upper fertilization thresholds, suggested average application rates of 194 kg N ha^−1^, 105 kg P_2_O_5_ ha^−1^, and 157 kg K_2_O ha^−1^, which align well with the nutrient requirements of rice. Additionally, substituting organic N for chemical N is an effective approach to conserve chemical fertilizer N, potentially reducing chemical N usage by 20%−40%. Utilizing slow-release N is also a favorable option to enhance N use efficiency and optimize N balance. This study offers valuable insights into the development of fertilization restriction indicators, aiming to achieve a delicate balance between environmental impact and agricultural productivity through the adoption of balanced fertilization rates and utilization of organic residues.

## 1 Introduction

Effective nutrient management is pivotal for achieving high crop yields while minimizing environmental impacts. The precise alignment of nutrient supply with crop requirements is essential to prevent nutrient losses and avoid yield penalties due to the under-application of fertilizer. Conversely, excessive fertilizer use can lead to yield stagnation, nutrient leaching, and environmental degradation (Penuelas et al., 2023). Accurately predicting fertilizer requirements is challenging, often leading to fertilizer misuse where applications exceed plant needs to ensure high yields (Ge et al., 2024). The overuse of chemical fertilizers in China has been a persistent issue, with usage rising from 12.7 million tons in 1980 to 50.2 million tons in 2023 (National Bureau of Statistics of China, 2024). Farmers’ reliance on historical experience for determining fertilizer rates contributes to unbalanced fertilizer applications, particularly the excessive use of nitrogen (N) and phosphorus (P) fertilizers (Cui et al., 2018). In China, the average N application rate is 305 kg N ha^–1^ yr^–1^, significantly higher than the global average of 74 kg N ha^–1^ yr^–1^ (Cui et al., 2018). Specifically, in rice production, the N fertilizer use of 209 kg ha^–1^ exceeds the global average by 90% (Chen et al., 2014). This surplus of N has led to various environmental issues, including soil degradation (Guo et al., 2010), nitrate leaching (Diaz and Rosenberg, 2008), and nitrous oxide emission (Zhang et al., 2016). Similarly, the overuse of P fertilizer poses a threat through eutrophication with 69% of P entering watercourses from agricultural sources (MEP, 2010). Given the escalating cost of fertilizers, quantifying incentives to reduce input usage becomes increasingly valuable for addressing potential imbalances faced by farmers. Developing an effective fertilization strategy is among the most critical decisions in agronomy, impacting not only on-farm expenses but also environmental outcomes that may not have been intended.

The trade-off between enhancing crop yield and mitigating environmental risk is a critical concern for researchers and policymakers alike, particularly regarding the optimal fertilization rate. Various methods based on plant or soil tests have been developed and implemented globally to recommend suitable fertilizer application rates (Dermawan et al., 2024; van Doorn et al., 2024; Colaço et al., 2024). However, plant tests, such as those using leaf color charts and chlorophyll meters, are inadequate for making decisions regarding starter fertilizer. Soil sampling to ascertain the amount of mineral N is a widespread approach in Europe and North America for adjusting N fertilization (Valkama et al., 2013). Nevertheless, this method is unreliable for determining the readily available N amount for rice crops due to the dynamic nature of N transformations in submerged soil, complicating sampling and analysis (Xu et al., 2019). Nutrient budgets and balances, principal agro-environmental indicators, provide abundant information on nutrient use efficiency, soil fertility, and the potential environmental impacts (Organization for Economic Co-operation and Development, OECD, 2001; Ju and Zhang, 2017). These tools have been used extensively for optimizing fertilizer recommendations and improving policymaking over the past decades (Buresh et al., 2010). Nutrient balance, the difference between the sum of inputs and outputs within an agricultural system, helps identify whether a soil nutrient is present in surplus or deficit. Compared to a full nutrient balance that considers many additional factors, a partial nutrient balance, determined by the difference between fertilizer input and crop removal, is simpler to implement and more cost-effective. Furthermore, calculating a partial nutrient balance is more precise than a full nutrient balance, which typically involves considerable uncertainty, especially when parameters are used to estimate input and output pathways on a large scale (Eurostat, 2023; Karimi et al., 2020). The partial nutrient balance is also a reliable indicator of some critical components of sustainability and is important for decision support in terms of soil fertility management (Ludemann et al., 2024). Despite its frequent use as a sustainability indicator for agricultural systems, there is scarce reporting on estimating fertilization thresholds based on partial nutrient balance.

The integration of improved mineral fertilizer management and organic residue recycling is pivotal for achieving long-term nutrient balances in agricultural soil (Ingwersen et al., 2024). This approach not only optimizes mineral nutrient input but also enhances nutrient use efficiency, thereby contributing to sustainable agricultural practices. Recycling crop residues like crop straw and applying organic fertilizers to fields is increasingly becoming a common practice globally as an effective form of conservation agriculture (Tang et al., 2024). In China, crop residue management plays a crucial role in maintaining soil health and nutrient cycles. With an estimated production of crop residues is 925 million tons of crop residues annually (Li et al., 2016), the country holds substantial potential for nutrient recycling through straw return to fields. Rice straw constitutes a significant portion of the total nutrient resources, accounting for 33.1% (Song et al., 2018). The importance of potassium (K) in crop straw is noteworthy, as most of the K is sequestered in the straw rather than the grain (Shukla et al., 2024). A negative K balance of up to -362 kg K ha^−1^ was observed where straw was removed, whereas a surplus of 29 kg K ha^−1^ was found in treatments where straw was returned to the field (Whitbread et al., 2003). Meta-analyses by Huang et al. (2013) and Ding et al. (2018) indicated that crop residue retention significantly increased rice yield by approximately 5%. Additionally, the application of organic fertilizers, such as farmyard manure, composts, and commercial manure, can further enhance nutrient use efficiency and soil health. Reports indicated that reduced applications of P and K following manure application did not decrease rice yield, and treatments with added manure resulted in more positive nutrient balances (Saha, 2007). Maintaining soil organic matter levels is crucial for sustainability, and organic fertilizers play a significant role in this process. However, the current rates of crop residue and manure return to fields in China are relatively low, with only 15-60% of crop residue (Li and Jin, 2011) and 30–65% of manure recycled (Zhang et al., 2010). Therefore, emphasizing the importance of these practices in sustainable agriculture is necessary to optimize nutrient management and promote environmental stewardship.

Advancing sustainable agricultural production necessitates improved management practices at the field scale and the implementation of effective policies at the regional scale. The announcement of the Chinese government’s policy of “Zero Growth of Chemical Fertilizer Use by 2020” has increased the demand for political decisions that balance agronomic-economic benefits with environmental consequences. Nutrient balance has been employed by policymakers as an agro-environmental indicator to raise awareness about nutrient use efficiency. However, there is a scarcity of studies providing explicit fertilizer recommendation thresholds at the national scale. To bridge this knowledge gap, a comprehensive synthesis of research was performed using existing data from multi-year and multi-site studies. The objectives were to: (1) propose a method for estimating the agro-environmentally allowed ranges of N, P, and K fertilization based on nutrient balance and rice yield results; (2) quantify the potential of straw return to reduce chemical fertilizer input; and (3) investigate the response of N balance to the substitution of conventional N with organic fertilizer and slow-release N fertilizer.

## 2 Materials and methods

### 2.1 Database

The database was created using both published and unpublished data from major rice-growing regions in China. The published data were sourced from a comprehensive review of peer-reviewed papers from 2000 to 2018, utilizing the China Knowledge Resource Integrated Database and Web of Science. The unpublished data came from on-farm experiments conducted by the International Plant Nutrient Institute (IPNI) China Program and our research team. The search included terms like rice yield, nutrient uptake, controlled or slow-release fertilizer, organic substitution, and straw return. Papers meeting the following criteria were selected: (1) conducted under field conditions in China; (2) availability of data on fertilizer rate, rice yield, and nutrient uptake data; and (3) estimation of fertilization thresholds using common chemical fertilizers, excluding high-efficiency fertilizers, organic fertilizers, and straw return techniques.

**Figure 1:**
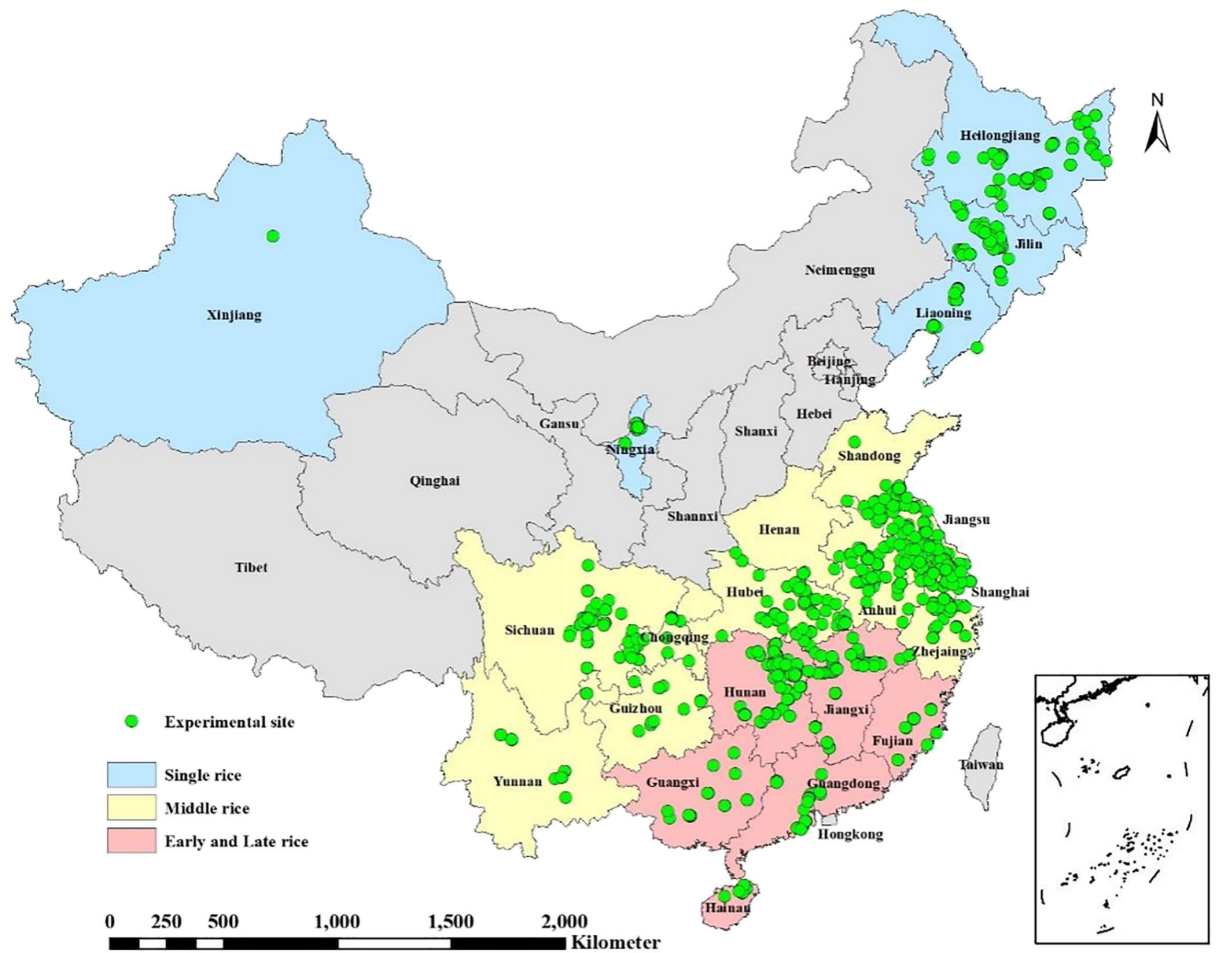
Geographic locations of experiments for four rice types in China.

The final database included 580 studies, with 422 from published papers. These studies were categorized into four datasets based on the rice planting season: (i) 99 studies in the single rice dataset (mono-season rice), primarily distributed in Northeast and Northwest China; (ii) 321 studies in the middle rice dataset (summer-season rice rotated with winter-season upland crops, such as winter-wheat or oil rape), primarily distributed in the middle and lower reaches of the Yangtze River and Southwest China; (iii) 123 studies in the early rice dataset (double-season rice cropping systems with early rice rotated with late rice), primarily distributed in the middle and lower reaches of the Yangtze River and South China; and (iv) 137 studies in the late rice dataset (double-season rice cropping systems with early rice rotated with late rice), shares the same distribution area as the early rice. The distribution of the four rice types and experimental sites is depicted in **Figure 1**.

### 2.2 Partial nutrient balance

The soil partial N, P and K balance (N_balance_, P_balance_ and K_balance_, respectively) was calculated as described by OECD (2001):


(1) $${\text{Nutrient}}_{\text{balance}}={\text{Nutrient}}_{\text{input}}-{\text{Nutrient}}_{\text{output}}$$

where *Nurient_input_* is the nutrients of N/P_2_O_5_/K_2_O applied as fertilizers and manure (kg ha^−1^), and *Nurient_output_* is the nutrients of N/P_2_O_5_/K_2_O accumulated in the grain and/or straw (kg ha^−1^) and removed from fields.

The exclusion of certain nutrient inputs and losses in calculating nutrient balance is crucial for assessing soil health and agricultural sustainability. In our calculation, we deliberately omitted the consideration of nutrients gained through biological N fixation, atmospheric deposition, and irrigation, as well as losses due to ammonia volatilization, denitrification, runoff, and leaching. A negative nutrient balance indicates that the removal of nutrients from the soil exceeds its replenishment, leading to soil nutrient depletion and a subsequent decline in crop productivity. Conversely, a positive balance with low nutrient use efficiency indicates that nutrients are partially accumulating in the soil, potentially posing risks of nutrient loss to the broader environment due to excessive application.

### 2.3 Statistical analysis

Following the data tests for normal distribution and equal variance, relationships between N_balance_, P_balance_ or K_balance_ and N, P or K rates were fitted using a least-squares linear regression:


(2) Y=ax+b

where Y is the soil partial nutrient balance for N, P or K; x is the N, P or K fertilizer rate; a is the fitted constants and b is the corresponding balance value without N, P or K fertilizer application.

The statistical model used to describe relationships between the yield and the N rate was a unitary quadratic model:


(3) $$\text{Y}=\text{Y}0+\text{ax}+{\text{bx}}^{2}$$

where Y is the rice yield; Y0 is the yield at N = 0; a and b are fitted constants; and x is the N rate.

A paired t-test was conducted to compare the N_balance_ between the organic substitution group and the conventional fertilization group using SPSS version 21 software. Means of different levels of some explanatory variables within the organic fertilizer groups were compared using the least significant difference test (LSD at 0.05 level of probability) by a one-way ANOVA analysis.

### 2.4 Principles for the thresholds of fertilization rates

The benchmarks for assessing nutrient balance are indeed intricate and subject to fluctuation, owing to the complex interplay of factors such as agroecosystem type, climate, soil type, and environmental conditions. The overarching goal of fertilization should encompass both the total nutrient demand for food security and the minimization of environmental impacts. To address these challenges, principles based on partial nutrient balance have been proposed to determine the lower and upper threshold of N, P, and K fertilizer application rates. The low limit of threshold, i.e., the minimum rate, is predicated on the principle that fertilizer inputs should at least match the nutrients removed by the crop. This threshold corresponds to the nutrient rate at which the partial nutrient balance is zero (i.e., x value at Y=0 simulated based on Equation 2), meaning that nutrient input is precisely balanced with output (crop uptake). In this case, the least nutrients are possibly lost to the environment according to the positive relationships between partial nutrient balance and nutrient losses (Mclellan et al., 2018). However, the minimum fertilization strategy heavily depends on the indigenous soil stocks, as a significant portion of fertilizer nutrients can be lost from soil-plant systems through mechanisms such as ammonia volatilization and nitrification-denitrification processes. Strategies that solely focus on crop removal without accounting for indigenous soil nutrient supply are inefficient for sustainable high-yield production systems. Though a nutrient surplus is generally discouraged due to the heightened risk of nutrient loss, a certain surplus based on partial nutrient balance is permissible to offset fertilizer losses and the depletion of indigenous soil nutrients. This allowance acknowledges the reality that not all fertilizer nutrients are utilized by the crop and are not accounted for in the partial nutrient balance calculation. The upper limit of the threshold, i.e., the maximum fertilization rate, takes into consideration the issues of nutrient losses, nutrient availability, and the maintenance of soil fertility. It is defined as the fertilizer rate when a partial nutrient balance is equal to nutrient supply from the soil (i.e., the absolute value of the Y-intercept based on Equation 2), as determined from plots without N, P, or K fertilization. The partial nutrient balance principle ensures that fertilizers are applied efficiently, minimizing environmental impacts while maximizing crop productivity.

The N nutrient is pivotal in influencing crop yield. Therefore, the grain yield level serves as another key indicator for determining N fertilizer application rates. The concept of ‘attainable yield’ is central to this approach, representing the highest yield achievable under current farming conditions, modeled through quadratic equations (Equation 3). van Wart et al. (2013) observed that in China, the 5-year average yields of rice tend to stabilize once the average farm yields reach 82% of the attainable yield. Similarly, Laborte et al. (2012) noted that the average rice yields in Southeast Asia can approach 83% of the attainable yield. In this context, our study adopts a practical yield goal range of 85% to 100% of the attainable yield. To operationalize this, the minimum and maximum fertilizer rates are determined by identifying the N rates at which the yield equals 85% and 100% of the attainable yield, respectively, based on Equation 3. When the N rate corresponding to a zero N_balance_ is even lower than the N rate under 85% attainable yield, the lower threshold of N rate is adjusted to match the latter; and the N rate under 100% attainable yield becomes the upper threshold if it is less than that the rate calculated with N_balance_ at N supply from the soil.

The relationship between rice yield and P or K rate is relatively weak, making it crucial to fine-tune fertilization based on soil nutrient status and crop demand. Therefore, the determination of P and K thresholds in fertilization strategies is grounded in the principle of partial nutrient balance. This principle aims to optimize the use of P and K fertilizers by considering the existing soil nutrient status and the crop’s requirements. The principle of partial P or K balance, akin to that of, determines the lower threshold of P and K rate as the point where the partial nutrient balance is zero, and determines the upper threshold when the partial nutrient balance equals P or K supply from the soil without P or K fertilization. In fertilization practice, the reference level for P and K balance could be adjusted according to the soil P and K status, being larger when soil P and K are low and smaller when they are high (Oenema et al., 2003). By balancing nutrient inputs with outputs and considering soil nutrient status, this method enhances agricultural sustainability, crop yields, and environmental stewardship.

## 3 Results

### 3.1 The limited rate of N, P and K fertilizer in rice

**Figure 2:**
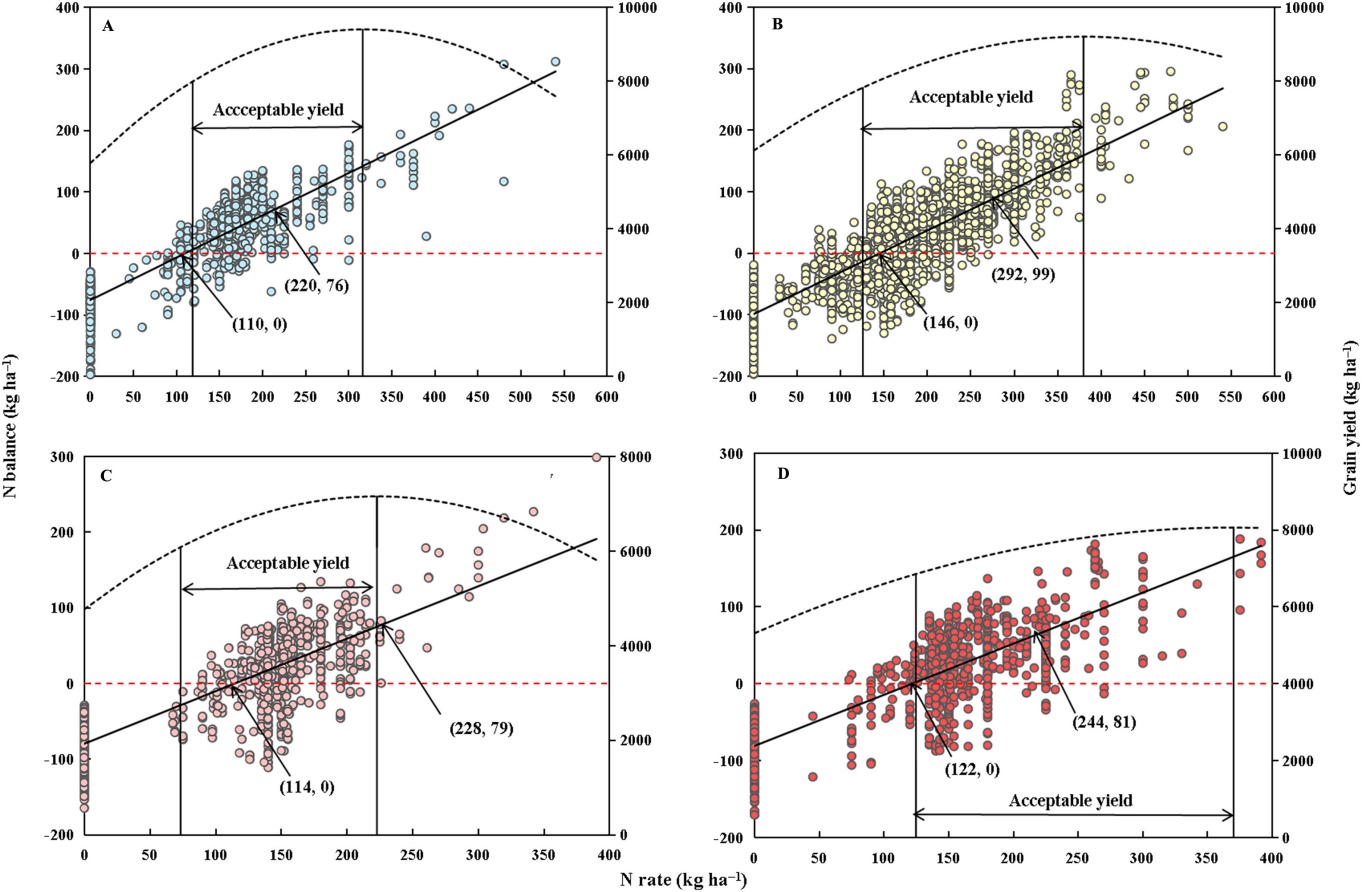
Relationships between N rate and N_balance_ and between N rate and grain yield for single- **(A)**, middle- **(B)**, early- **(C)**, and late-season rice **(D)**, respectively. The curve is the yield (data points not shown), and the straight line and data points are the N_balance_. The values in parentheses are the N rates and their corresponding N balance values. The acceptable yield ranges from 85% to the highest yield.

Rice yield and N_balance_ were individually regressed against the N rate (as depicted in **Figure 2**), with the associated parameters delineated in **Table 1**. The results showed a significant positive linear relationship between the N rate and N_balance_, alongside a quadratic relationship linking the N rate and rice yield. Soil inherently supplied 76.4−99.4 kg N ha^−1^ while the peak attainable yield spanned from 7156 to 9297 kg ha^−1^ across four rice types. As illustrated in **Figure 2**, adhering solely to the dual principles of N balance (i.e., N_balance_ value is zero, and the N surplus equals the N uptake contributed by the soil) cannot meet the demand of acceptable grain yield (85% of the highest yield to the highest yield) in some cases. Consequently, integrating considerations of N balance and rice yield, the optimal N rate spectrum ranged from 119 to 220, 146 to 292, 114 to 223, and 124 to 242 kg N ha^−1^ for single, middle, early, and late rice, respectively, corresponding to 85−97%, 88−99%, 92−100%, and 85−96% of the respective highest yields.

**Table 1:** Parameters and fittings of the linear and quadratic models that describe the relationship between N_balance_ and N rate, and the relationship between rice yield and N rate.

Rice type	Linear model (Y = ax + b)			Quadratic model (Y = Y0 + ax + bx^2^)				n
	a	b	R^2^	a	b	Y0	R^2^	
Single	0.694	−76.4	0.763^**^	23.764	−0.0384	5621	0.315^**^	1081
Middle	0.681	−99.4	0.799^**^	16.679	−0.0229	6089	0.352^**^	3472
Early	0.692	−78.9	0.656^**^	21.524	−0.0485	4768	0.260^**^	1123
Late	0.663	−80.2	0.688^**^	15.000	−0.0204	5301	0.251^**^	1116

R^2^, coefficient of determination.

n, number of observations.

^**^ represents significance at p< 0.01.

**Figure 3:**
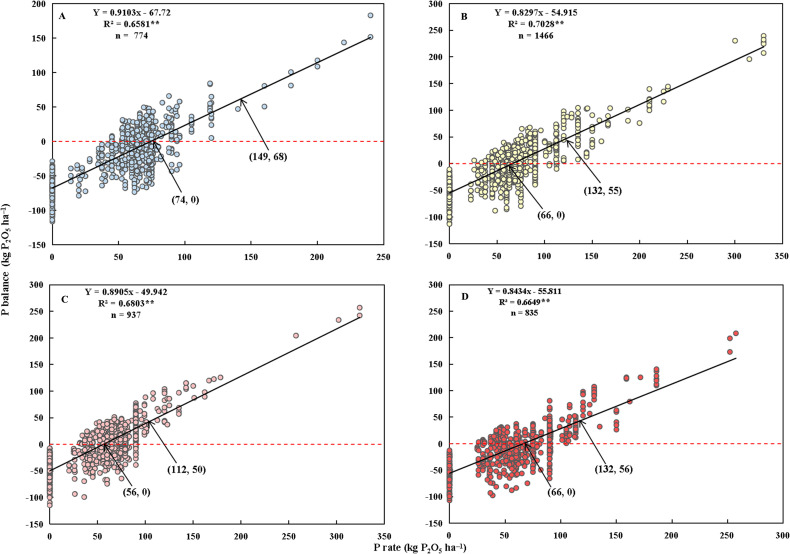
Relationships between P rate and P_balance_ for single- **(A)**, middle- **(B)**, early- **(C)**, and late-season rice **(D)**, respectively. The values in parentheses are the P rates and their corresponding P_2_O_5_ balance values. R^2^ is the coefficient of determination. n is the number of observations. ** represents significance at *p*< 0.01.

The relationship between P application and P_balance_ was fitted using a linear model for each of the four rice types (as depicted in **Figure 3**). A significant positive correlation was observed, with P application accounting for 66% to 80% of the variability in P_balance_. To minimize P_balance_ in the soil while ensuring crop requirements are met, the minimum P input should be in the range of 56−74 kg P_2_O_5_ ha^−1^ across the four rice types, considering crop removal. Given that P losses due to leaching and runoff constitute a relatively small percentage of total P fertilizer, the minimum P input appears sufficient for crop demands. However, the bioavailability of both fertilizer and soil P in the current season remains a concern. According to the linear model, the soil contributes approximately 50−70 kg P_2_O_5_ ha^−1^ P uptake. Consequently, to restore soil P depletion and maintain soil available P at an optimal level, the maximum P input can extend to 112−149 kg P_2_O_5_ ha^−1^, serving as the upper limit for P thresholds.

**Figure 4:**
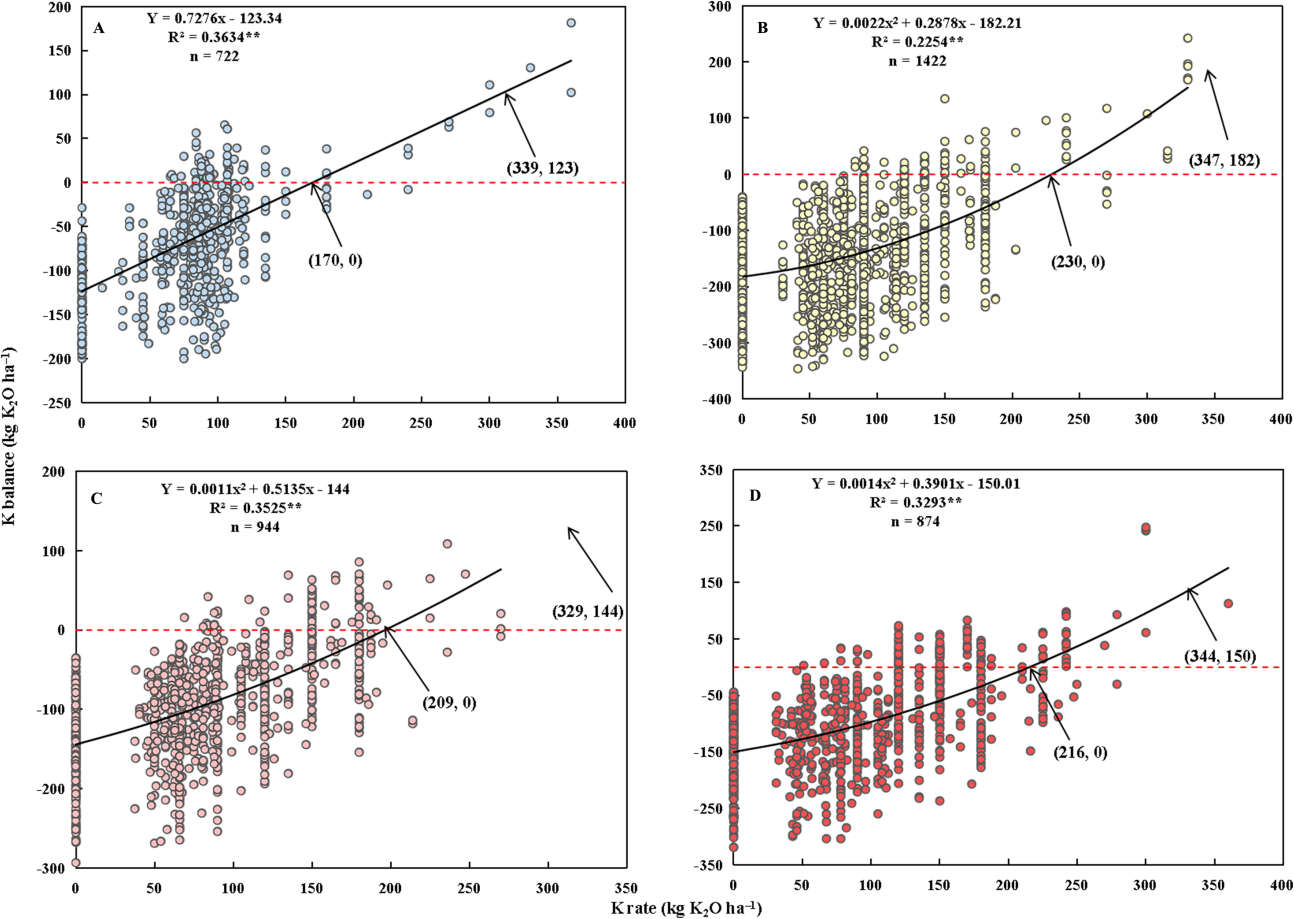
Relationships between K rate and K_balance_ for single- **(A)**, middle- **(B)**, early- **(C)**, and late-season rice **(D)**, respectively. The values in parentheses are the K rates and their corresponding K_2_O balance values. R^2^ is the coefficient of determination. n is the number of observations. ** represents significance at *p*< 0.01.

Similarly, K_balance_ was fitted to the K rate using a linear model for single rice, akin to the process employed for N and P. However, for middle, early, and late rice, the quadratic model provided better fits than the linear model (as shown in **Figure 4**). Positive associations were identified between the K application and K_balance_, with the K application explaining 23% to 36% of the variation in K_balance_. To sustain an essential K balance, the recommended K fertilizer applications are 170, 230, 209, and 216 kg K_2_O ha^−1^ for early, middle, early, and late rice, respectively. Based on the models, the soil contributed roughly 120−180 kg K_2_O ha^−1^ towards K uptake. These contributions are utilized as benchmarks for maximum K surplus. Thus, the upper K thresholds were 339, 347, 329, and 344 kg K_2_O ha^−1^ for early, middle, early, and late rice, respectively.

### 3.2 Effect of straw return on nutrient balances

The analysis presented was indeed based on scenarios involving rice straw removal, leading to estimated ranges of fertilization requirements, particularly for K, that were somewhat elevated compared to expectations. However, under the hypothesis of returning rice straw to the fields, there would be notable enhancements in the soil’s N, P, and K balances. Given the scenario of acceptable grain yield targets ranging from 6082 to 9297 kg ha^−1^, nutrient balances under straw return were recalculated to gauge the adjustments in fertilization rates relative to straw removal scenarios. Results showed that on average, returning straw to fields can increase N, P, and K balances by 52.0 kg ha^−1^, 25.8 kg P_2_O_5_ ha^−1^, and 182.6 kg K_2_O ha^−1^, respectively. This suggests that fertilizer nutrients can be partially offset by the practice of straw return (**Table 2**). Specifically, for single, middle, early, and late rice under straw return, N_balance_ can be increased by 46.3, 57.0, 42.6, and 53.9 kg ha^−1^, respectively; P_balance_ can be increased by 32.9, 21.8, 20.7, and 27.9 kg P_2_O_5_ ha^−1^, respectively; and K_balance_ can be increased by 136.3, 233.3, 172.4, and 188.4 kg K_2_O ha^−1^, respectively. This shows that straw return has a considerably more significant impact on K fertilization management than on N and P management.

**Table 2:** Comparisons of nutrient balances between rice straw removal and return under acceptable yield goals.

Rice type	Balances under straw removal (kg ha^−1^)			Balances under straw return (kg ha^−1^)			Increases in balance (kg ha^−1^)			Acceptable yield (kg ha^−1^)
	N	P_2_O_5_	K_2_O	N	P_2_O_5_	K_2_O	N	P_2_O_5_	K_2_O	
Single	41.3 ± 3.5	-24.3 ± 2.3	-89.9 ± 5.2	87.7 ± 3.5	8.6 ± 2.1	46.4 ± 3.0	46.3 (249)^a^	32.9 (207)	136.3 (194)	7903−9297
Middle	20.5 ± 2.4	-6.0 ± 2.3	-184.4 ± 5.1	77.5 ± 2.5	15.8 ± 1.8	48.9 ± 2.4	57.0 (747)	21.8 (432)	233.3 (421)	7757−9126
Early	11.3 ± 3.5	-8.3 ± 2.9	-128 ± 5.5	53.9 ± 3.5	12.3 ± 2.8	44.4 ± 4.0	42.6 (293)	20.7 (160)	172.4 (157)	6082−7156
Late	16.7 ± 3.9	-19.7 ± 3.2	-128.9 ± 8.2	70.6 ± 3.9	8.2 ± 3.1	59.5 ± 5.5	53.9 (209)	27.9 (174)	188.4 (176)	6850−8058
Total	21.7 ± 1.6	-14.6 ± 1.4	-132.8 ± 3.3	73.7 ± 1.7	11.2 ± 1.2	49.8 ± 1.7	52.0 (1498)	25.8 (973)	182.6 (948)	6082−9297

Values following the “±” are standard errors.

a Numbers of observation in parentheses.

### 3.3 Effect of organic substitution on N_balance_

**Figure 5:**
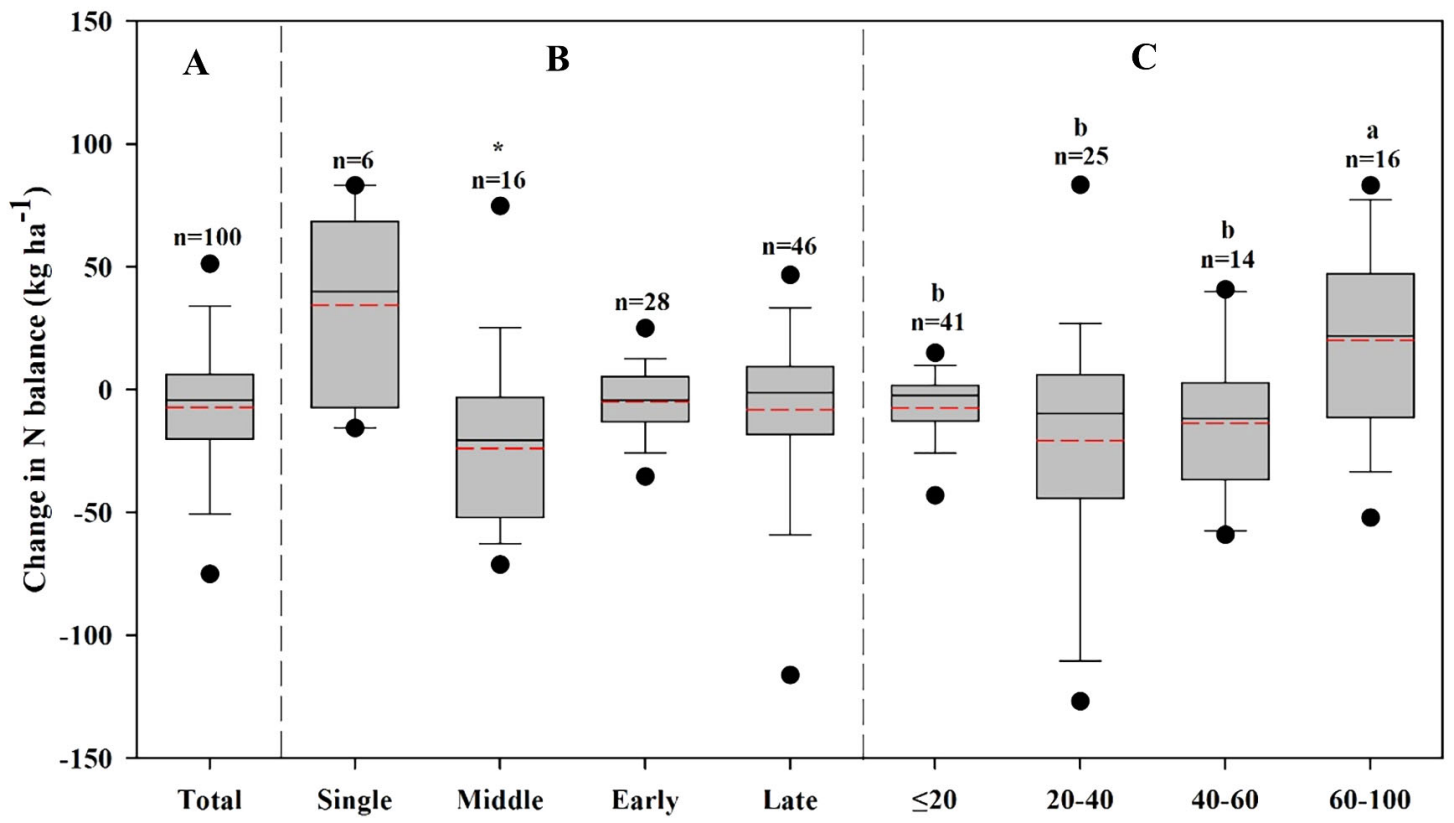
Changes in N_balance_ under organic N substituting inorganic N relative to inorganic N alone across the total dataset **(A)**, different rice type **(B)**, and the percentage of organic N in total N **(C)**. “*” represents a significant change in N balance at *p*< 0.05. Different lowercase letters indicate significant difference (*p*< 0.05) within the category. n is the number of observations.

Overall, substituting chemical fertilizer N with organic fertilizer (10−100%) did not result in a significant change in N_balance_ when compared to the application of chemical fertilizer alone (**Figure 5**). However, the changes in N_balance_ were statistically different between different rice types and the substituted percentage of organic fertilizer. An increase of 34.5 kg ha^−1^ in N_balance_ was observed under single rice cultivation, while a significant decrease of 20.7 kg ha^−1^ was noted under middle rice cultivation. This suggests that the substitution of organic fertilizer led to differences in N uptake, given that the total N rates of chemical and organic fertilization were the same. When the percentage of organic substitution ranged from 20 to 40%, the decrease in N_balance_ was the lowest, indicating an optimal percentage of organic N. A significantly higher increase of 20.2 kg ha^−1^ occurred when the percentage of organic N exceeded 60%, implying a negative effect on N uptake or rice yield. Exaggerated percentages of organic N should be avoided.

### 3.4 Effect of slow-release N fertilizer on N_balance_

**Figure 6:**
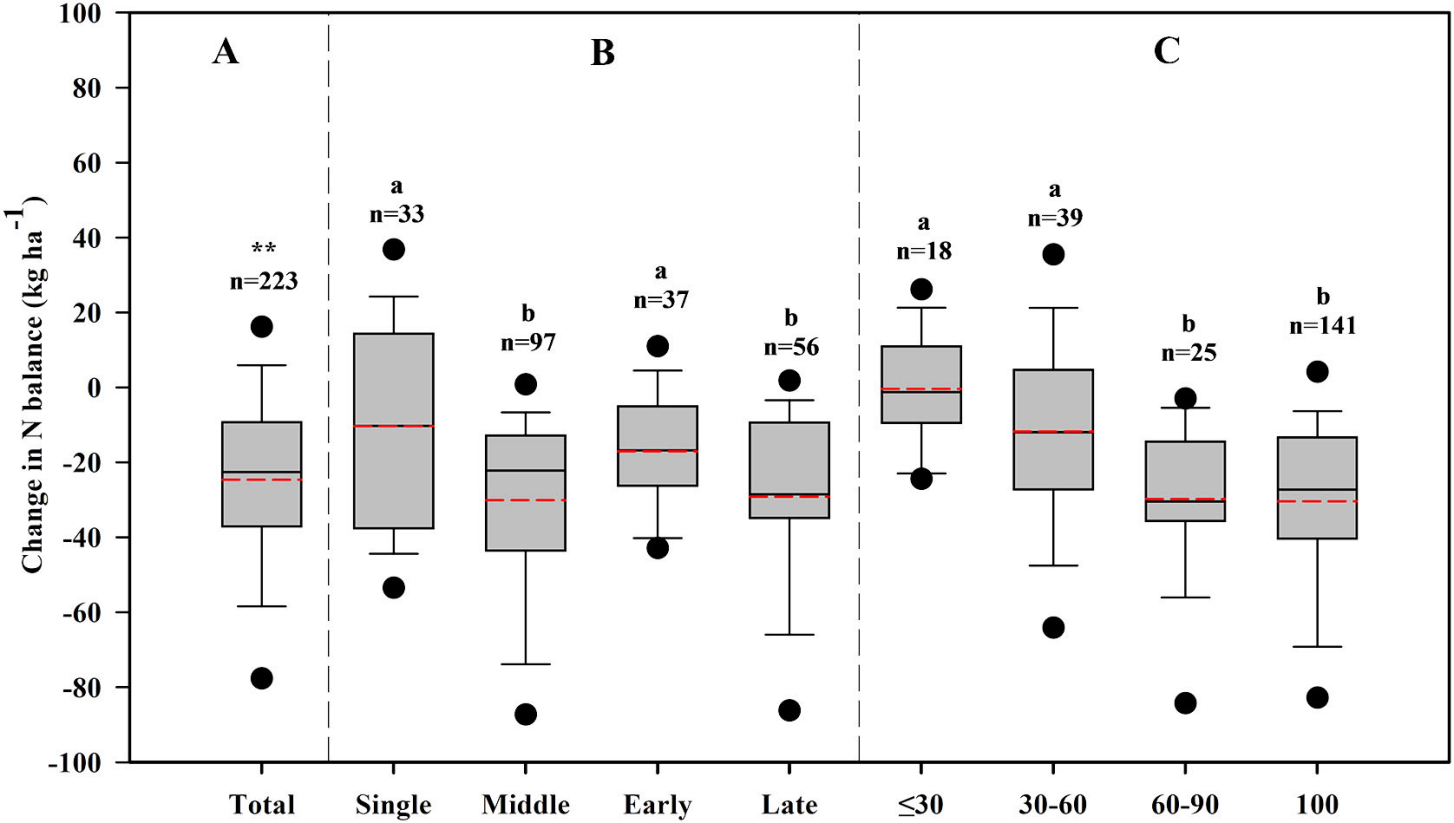
Changes in N_balance_ with application of slow-release N fertilizer relative to conventional N fertilizer across the total dataset **(A)**, different rice type **(B)** and the percentage of slow-release N in total N rate **(C)**. “**” and “*” represent significant changes in N balance at *p*< 0.01 and *p*< 0.05, respectively. Different lowercase letters indicate significant differences (*p*< 0.05) within the category. n is the number of observations.

On average, there was a significant decrease (−24.3 kg ha^−1^) in N_balance_ when conventional N was substituted with varying percentages (10−100%) of slow-release N (**Figure 6**), indicating more N was taken up by the rice plants. This decrease in N_balance_ was more pronounced under middle and late rice compared to those under single and early rice. The percentage of slow-release N in total N applications significantly influenced N_balance_. A negative correlation was observed between the percentage of slow-release N in total N and the decrease in N_balance_. Decreases in N_balance_ were notably larger when the percentage of slow-release N exceeded 60%. Changes in N_balance_ with slow-release N fertilizer primarily depended on changes in rice yield. Applying slow-release N to middle and late rice at a high percentage led to a relatively higher yield.

## 4 Discussion

### 4.1 The thresholds of N/P/K fertilization in rice fields

Maintenance of soil fertility by an external source such as commercial fertilizer is essential for continued attainment of high target yields as crop removal depletes finite soil reserves of N, P, and K over time. However, certain fertilizer management practices in intensively managed agriculture systems fail to link soil nutrient balance with yield level, leading to unbalanced applications of N, P, and K, which can cause yield stagnation or declines (Wang et al., 2024). In our study, the estimation of N rates based on N balance meets the requirement of attainable yield goals. The maximum N surplus is set at a value equal to the soil contribution to crop N uptake (averaged 84 kg N ha^−1^), under which the N rate may serve as a reference of the upper limit of the N threshold. Considering the priority of yield and the current fertilization techniques in China with N losses up to 40−50% (Gu et al., 2015), a N surplus less than soil consumption is essential for building soil fertility and avoiding over-surplus to exacerbate N loss. Zhang et al. (2019) established N surplus benchmarks for single cropping systems ranging from 40 to 100 kg N ha^−1^ in China. Therefore, on the promise of a high yield level, the lower and upper thresholds of N rates given by the present study accommodate the dual needs of minimizing environmental losses and maintaining crop productivity. Ju et al. (2004) estimated the rational range of N rate for rice in China to be 150−250 kg ha^−1^ based on yield levels of 6500−8500 kg ha^−1^. The upper limit they suggested is similar to our result (averaged 244 kg N ha^−1^), while the lower limit is slightly higher than ours (averaged 126 kg N ha^−1^) due to their emphasis on yield and disregard for the high indigenous soil nutrient supply. Zhu (2006) proposed a regional mean optimal rate of fertilizer N for rice at the Middle and Lower Reaches of the Yangtze River, recommending a relatively narrow range of 190−200 kg N ha^−1^, covering partial cultivation areas of middle, early, and late rice. Their recommended rates are encompassed in our findings and may be more suitable in a specific region, but not on a national scale. Differences in N thresholds were observed among different rice types (early, middle, late, and single-season rice) due to variations in soil, climate, rotation crops, and varieties (Xu et al., 2015, 2016). For example, considering higher N losses with alternative drying and wetting, Ju and Gu (2014) suggested an allowed surplus of 100 kg N ha^−1^ and a maximum N rate of 300 kg N ha^−1^ for rice in a wheat-rice rotation system, which aligns closely with the upper estimation for middle rice in our study (292 kg N ha^−1^). Coincidentally, rice growers in the Tai Lake Catchment (dominated by middle rice) applied approximately 300 kg N ha^−1^ on average (Jiang et al., 2012), implying a substantial potential for N reduction. Current nutrient balance status in different regions serves as another reference when recommending N rates. He et al. (2018) found that the highest and lowest N balance levels were observed in Southwest and Northeast China, respectively. Therefore, in regions with a low N balance level, applying more N, potentially nearing the upper threshold, can be beneficial, while excessive N should be avoided in the regions with a high N balance level. If soil N pools are greatly depleted in one season, maintaining sustainable high yields in the subsequent seasons becomes challenging (Zou et al., 2024). Consequently, the N recommendation should be combined with the soil fertility assessments. Where indigenous N supply is abundant, N rates based on lower thresholds are suggested to maximize N use efficiency and minimize environmental risks. Conversely, in areas with lower soil fertility, N rates based on upper thresholds are suggested to guarantee high yields and replenish soil N consumption. It is crucial to recognize that neither the upper nor the lower threshold constitutes an optimal rate for fertilizer recommendation; instead, they provide an environmentally and agriculturally acceptable range within which site-specific fertilizer recommendations can be implemented. Moreover, careful management practices, such as deep application, spilt application, and high-efficiency N fertilizer, need to be conducted to improve N use efficiency and reduce N loss. Our results demonstrated that conventional N combined with slow-release N significantly decreased N_balance_ by increasing N uptake.

A rational application of P fertilizer is notable, given its widespread mismanagement in Chinese farmlands (Gu et al., 2024). According to the statistics conducted by Ma et al. (2018), the average net soil P surplus in China increased from 4.6 kg P ha^−1^ in 1980 to 42.1 kg P ha^−1^ in 2012 due to extensive P fertilizer application, and the accumulation of P in agricultural soils could theoretically sustain crop P demands for approximately 4.8–12.0 years. High native P-fixing capacity in acid soils (fixed by Al and Fe oxides) and alkaline soils (fixed by Ca) make P slowly available to plants. Nevertheless, lowland soils exhibit a greater capacity to supply P than upland soils due to reduced Fe (III) compounds under flooded conditions, which implies that excessive P application in rice fields can result in a lower P use efficiency (Dhillon et al., 2017). The present study estimated that the lower threshold of P fertilizer application for rice ranged from 56 to 74 kg P_2_O_5_ ha^−1^, and the upper threshold ranged from 112 to 132 kg P_2_O_5_ ha^−1^ across four rice types. The P uptake by rice was increased by only 9 kg P_2_O_5_ ha^−1^ when P fertilization increased from the lower to the upper limit. The P surplus was tentatively set at 20 kg P_2_O_5_ ha^−1^ per year for arable soils (Oenema et al., 2003), while our upper thresholds were estimated based on P surpluses of 50–68 kg P_2_O_5_ ha^−1^ per season. Furthermore, the P recovery efficiency was only averaged at 13.2% based on our database (data not shown). It has been reported that 100 kg ha^−1^ P surplus increased soil Olsen-P by 1.4–5.7 mg kg^−1^ under different soil types, and the risk of P leaching increased rapidly when soil Olsen-P exceeded 40 mg kg^−1^ (Li et al., 2011). Therefore, unless P is the most limiting factor in the soil, the upper level of P fertilization is not encouraged. Dobermann et al. (1996) suggested applying 46–57 kg P_2_O_5_ ha^−1^ for rice to maintain a good P balance in soil, which essentially matches the lower thresholds we proposed. Based on the condition of P surplus and the greater P-supply in flooded soil, we also recommend rice growers in China use P fertilizer referring to the lower thresholds.

Rice cultivation requires balanced nutrient management, including K, which is often overlooked despite its crucial role in crop yield and quality. The misalignment between the equivalent N and K uptake requirements in farmer’s practices and the actual needs of rice plants has led to widespread deficiencies in soil available K, exacerbated by high crop removal rates and unbalanced fertilization practices (He et al., 2015; Liu et al., 2017). Zhang et al. (2010) determined negative partial K balances in rice-based systems under long-term fertilization, ranging from 17 to 245 kg K ha^−1^ year^−1^, underscoring the need for adequate K supplementation. Recent studies demonstrated that applying K fertilizer significantly improved rice yield (Chen et al., 2024; Xu et al., 2023), indicating the importance of K in crop performance. When rice straw is included in K output, our study determined that large amounts of K fertilizer (averaged 206 kg K_2_O ha^−1^) need to be input to maintain essential K_balance_, even as high as 340 kg K_2_O ha^−1^ to offset K losses and replenish soil K consumption. However, while the lower K threshold precisely reflected the crop requirement, the upper thresholds of K fertilization may be overestimated when solely using soil contribution as the standard of maximum K surplus. The scarcity of research on K loss makes it challenging to establish a definitive standard for K_balance_, suggesting that using partial nutrient balance to estimate upper K thresholds is insufficient. A more refined method to assess K requirements is warranted. In addition, soil available K varies widely across China, with higher levels typically found in middle and early/late regions compared to single-rice regions (He et al., 2015) emphasizing the need for site-specific K fertilizer application. Given the growing recognition of K as a limiting factor in agricultural production, addressing K deficits has become a priority in recent years. Despite an increase in K fertilizer input from 0.4 million tons in 1980 to 9.4 million tons in 2010 (Liu et al., 2017), the vast amounts of K fertilizer needed to fully maintain soil K balance remain economically prohibitive for farmers due to the high cost of K fertilizer. Exploring strategies to enhance K use efficiency is therefore imperative.

### 4.2 Fertilizer rate changes with the addition of organic residues

The substantial expansion of Chinese agricultural production, characterized by increased crop yields and intensified livestock farming, has generated substantial volumes of animal wastes and crop residues, which are now recognized as significant nutrient reservoirs that can substitute for mineral fertilizers (Mu et al., 2024). The integration of mechanized farming and government-backed initiatives promoting crop straw return and organic fertilizer use has sparked a growing interest in optimizing the use of organic resources to offset mineral fertilizer application. Fertilization strategies are profoundly influenced by the addition of organic residues, such as crop residues and manure. The removal of rice straw from fields necessitates increased fertilizer inputs to maintain soil nutrient balance, particularly for K. Rice straw is distinguished by its high K content relative to other residues, averaging 22.7 g kg^−1^ in our database. The recycling of rice straw contributes to building a robust soil K pool, crucial for ensuring high rice yields and efficient K use efficiency. Our study showed that K_balance_ can be decreased by a large amount (182.6 kg K_2_O ha^−1^) with the return of rice straw, with a relatively lower but still significant reduction of 50.3 kg N ha^−1^ and 25.8 kg P_2_O_5_ ha^−1^. Although substituting substantial quantities of N fertilizer with straw is seldom practiced by farmers due to concerns about the high C/N ratio in straw causing rapid immobilization of soil mineral N, an optimal combination of chemical N and better N-spilt regimes can facilitate the release of straw N (Ding et al., 2019). In contrast, K is released from the straw considerably faster than the straw’s decomposition, primarily because K is predominantly present as K ions in vacuoles, enabling easy mobility and leaching (Chen et al., 2016; Murrell et al., 2021). Li et al. (2014) revealed that the decomposition of rice straw in flooded paddy soil can be divided into two main stages: an initial rapid phase from day 0 to 60, followed by a slower phase from day 60 to 110. The seasonal release of K from rice straw is approximately 85% (Liu and Li, 2017), contributing to an increase in soil exchangeable K supply and reserves (Han et al., 2024). However, straw return alone is insufficient to sustain soil K levels, necessitating supplementation with K fertilizer (Tan et al., 2007; Wang et al., 2010). Therefore, we recommend that rice growers refer to the upper thresholds as total nutrient rates and exclude N and K from returned straw as an optimized fertilization practice (i.e., N rate=244.8-50.3≈194 kg N ha^−1^; K rate=340.0-182.6≈157 kg K_2_O ha^−1^). According to the relationships in **Figure 2**, 194 kg N ha^−1^ rate can achieve high yield goals, and it agrees with the fertilization recommendations by other researchers. Technically, these reductions depend on how straw is managed; for example, optimizing management involves straw that needs to be well mechanically crushed before being incorporated into the soil and flooded during the winter. In recent years, with the increase in fertilizer K application combined with straw return, the K deficiency has been widely mitigated, and in some cases, soil K level changed from a deficiency to a surplus (Huang et al., 2007; Liu et al., 2017).

Manure stands out as a critical organic resource for numerous cropping systems, offering a potential avenue to curtail environmental pollution while sustaining crop yields in agricultural production (Ding et al., 2018; Smith et al., 2008). However, the extent to which rice nutrient demands can be met from organic sources remains uncertain. Our findings demonstrated that substituting chemical N fertilizer with manure can achieve almost a comparable N_balance_ status as the exclusive use of chemical fertilizers, suggesting that organic substitution does not adversely affect rice yields. Furthermore, the greatest reductions in N_balance_ were observed when the proportion of organic substitution ranged from 20-40%. This is attributed to manure not only enhancing crop yield but also increasing total N in the crop (Tirol-Padre et al., 2007; Duan et al., 2011). This aligns with our previous meta-analysis demonstrating that substituting chemical N with organic N maximized rice yield when the substitution proportion was approximately 30% (Ding et al., 2018). Another study corroborates this, showing that combining 30% organic N with 70% mineral N optimizes rice yield (Hou et al., 2011). Hence, it is feasible to decrease N fertilizer input by at least 30% while maintaining high and steady yields. However, a negative effect of organic substitution was observed in single rice cultivation, predominantly in cooler climates (northeastern and northwestern China) (Xu et al., 2016). The slow decomposition and mineralization rates of manure make it challenging to release as many available nutrients as in tropical and subtropical areas in the first season. Therefore, rice growers in single rice cultivation areas should exercise caution regarding the quantity and method of manure application. Beyond reducing fertilizer N input, manure application creates a substantial capacity for immobilizing mineral N applied to the soil, thereby mitigating environmental N loss (Bouwman et al., 2010; Smith et al., 2001; Zhou et al., 2016). Long-term manure application allows for the reduction or elimination of chemical P and K fertilizers while sustaining optimal grain yields (Duan et al., 2011). Although our present study did not involve substituting fertilizer P with manure, we posit that a considerable reduction in P fertilizer usage can be achieved through the addition of manure due to its high P concentration. According to our database (data not shown), the P concentration in manure is as high as 26.4 g P_2_O_5_ kg^−1^, which underscores its potential as a rich source of P. Li et al. (2011) reported that the manure application accelerated the increase of soil Olsen-P at a rate nearly 3 times higher than when an equivalent amount of P was applied solely as chemical fertilizer. This observation supports the notion that manure P can play an increasingly significant role as a substitute for P fertilizer, with an associated increase in P use efficiency. Therefore, determining the optimal proportion of mineral P combined with manure and the readily available P in manure is a critical area deserving of further investigation.

### 4.3 Study limitations

The complexity and challenges inherent in determining optimal fertilization rates for rice cultivation in China are significant, particularly due to the variability in yield responses to fertilization and the need to consider multiple factors such as the yield potential of rice varieties, climate, location, soil fertility, and management practices. While the method proposed can be used by farmers in practice, the specific thresholds of fertilizer N, P, and K given in the present study are more suitable for providing policymakers with a reference for restrictions on chemical fertilizer usage. The effectiveness of this method on a field scale is contingent upon an accurate assessment of soil nutrient status and an understanding of crop nutrient demands. Evaluating soil nutrient budgets with the partial nutrient balance may cause relatively large errors when great differences exist between ignored inputs and outputs. This mismatch between nutrient surplus and soil depletion underscores the importance of site-specific soil fertility in achieving high fertility levels and avoiding environmental consequences (Janssen and de Willigen, 2006). The current study acknowledges the influence of soil fertility on determining fertilizer rate but calls for more accurate indicators in future studies. Changes in fertilizer distribution and application techniques over time imply that the optimal fertilizer rates will evolve (Ladha et al., 2005). Incorporating increasingly optimized management practices into fertilization strategies was not addressed in the present study. In the case of straw removal, standing stubble remaining in the fields by the harvester can also contribute significant amounts of nutrients, which deserves further quantification. Overall, a systematic field validation based on our established fertilization criteria is deserved in the future.

## 5 Conclusion

This study proposed a systematic method for estimating the optimum ranges of N, P and K fertilizer for rice cultivation in China, which hinges on dual principles: nutrient balance and rice yield levels. By establishing lower and upper thresholds for N, P and K rates across four rice types, the method aims to delineate a balanced fertilization range beneficial for both farmers and policymakers. The lower fertilization limit, grounded in maintaining crop productivity while minimizing environmental pollution, serves as a baseline for sustainable agricultural practices. On the other hand, the upper limit, though currently lacking legislative backing akin to European Union standards, acts as a benchmark for fertilization regulations. Fertilization exceeding upper thresholds should be avoided as it can significantly escalate environmental costs, including nutrient leaching and greenhouse gas emissions. Optimizing fertilization recommendations involves the strategic application of slow-release N fertilizer, organic substitution, and straw return into the soil can markedly reduce the demand for K fertilizers and substantially decrease N and P requirements. The substitution of chemical N with organic fertilizers or slow-release N fertilizers improves N use efficiency, contributing to a more balanced N management strategy. This methodology is a valuable contribution to fertilization guidelines, not only for rice but also for other crops, fostering sustainable agricultural practices.

## Data Availability

The raw data supporting the conclusions of this article will be made available by the authors, without undue reservation.
